# Supplementary material for: Novel Bat Adenovirus Closely Related to Canine Adenoviruses Identified via Fecal Virome Surveillance of Bats in New Mexico, USA, 2020–2021
Source: Viruses. 2025 Oct 8;17(10):1349. doi: 10.3390/v17101349 (PMC12568287; doi:10.3390/v17101349)

Supplementary Materials

Figure S1: Serial dilution assay for limit of detection for pan-coronavirus RT-PCR (SARS-CoV-2 copies per reaction).

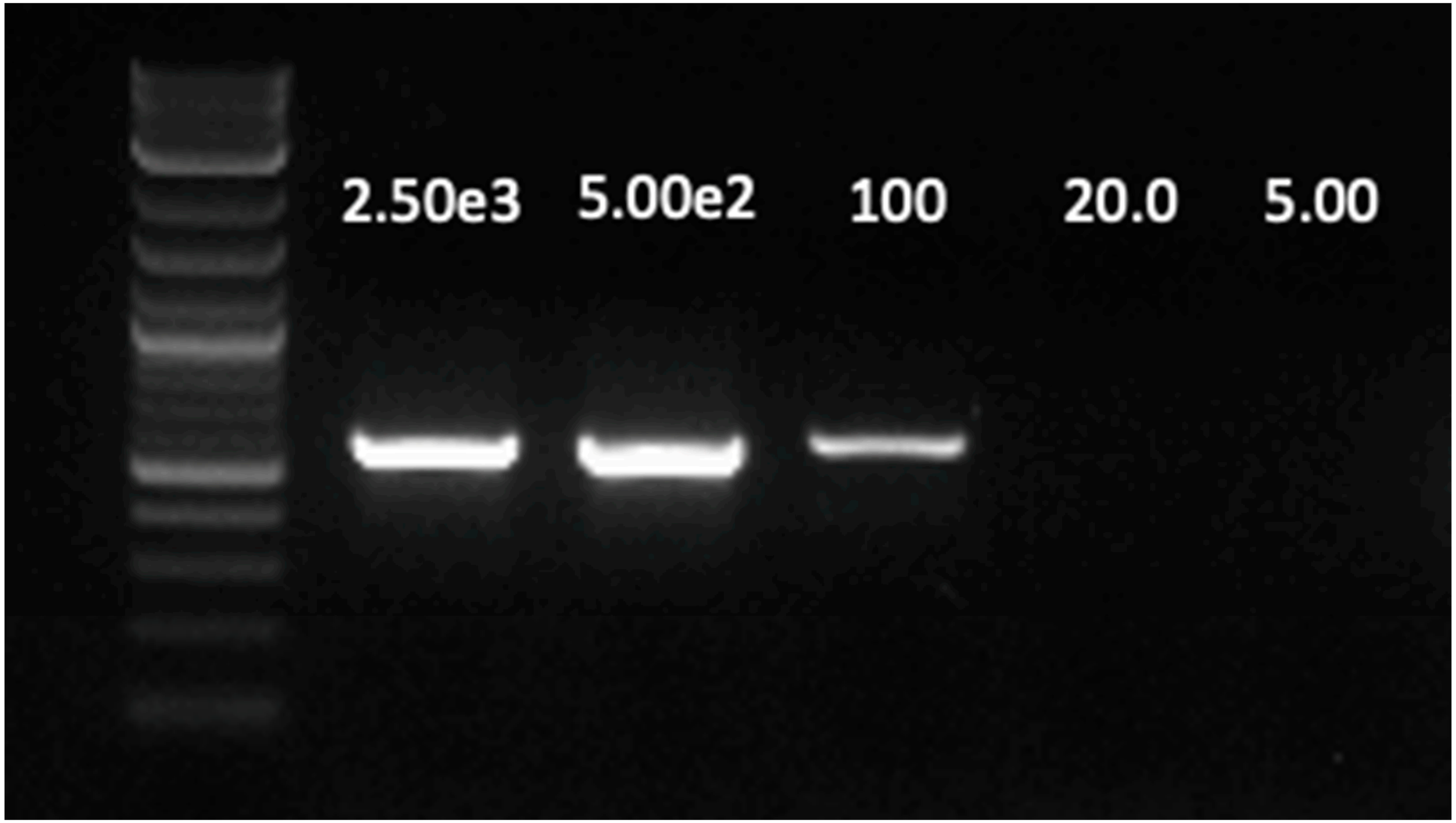

Figure S2: Maximum likelihood phylogenetic tree of adeno-associated viruses. The tree is outgroup rooted with snake adeno-associated virus. Taxon names are followed by GenBank accession numbers in parentheses. The taxon name of the virus identified in this study is in bold. Numbers beside nodes indicate bootstrap values (percent); scale bar indicates nucleotide substitutions per site.

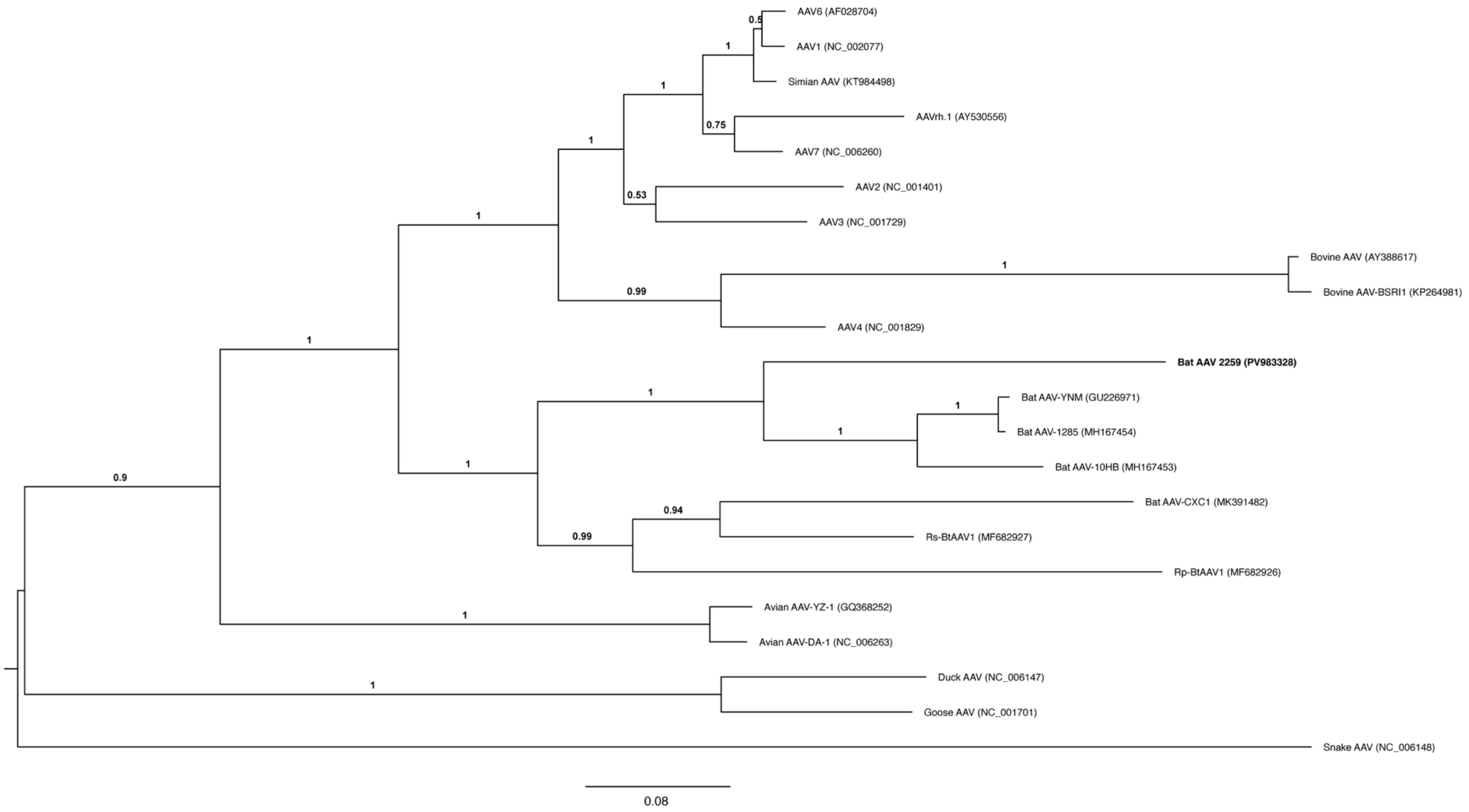

Figure S3: Maximum likelihood phylogenetic tree of astroviruses. The tree is outgroup rooted with turkey astrovirus. Taxon names are followed by GenBank accession numbers in parentheses. The taxon name of the virus identified in this study is in bold. Numbers beside nodes indicate bootstrap values (percent); scale bar indicates nucleotide substitutions per site.

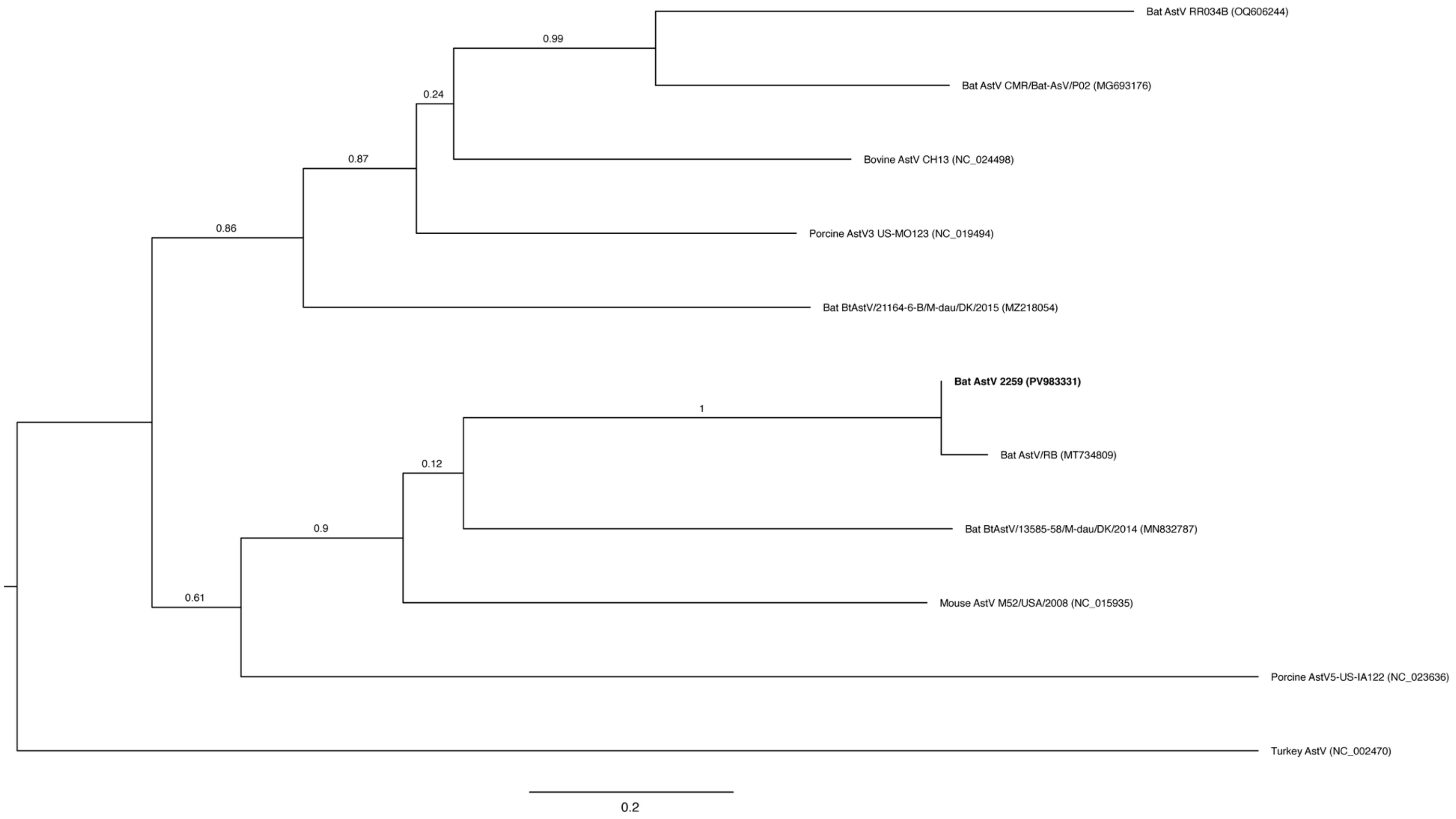

Figure S4: Maximum likelihood phylogenetic tree of genomoviruses. Taxon names are followed by GenBank accession numbers in parentheses. The taxon name of the virus identified in this study is in bold. Numbers beside nodes indicate bootstrap values (percent); scale bar indicates nucleotide substitutions per site.

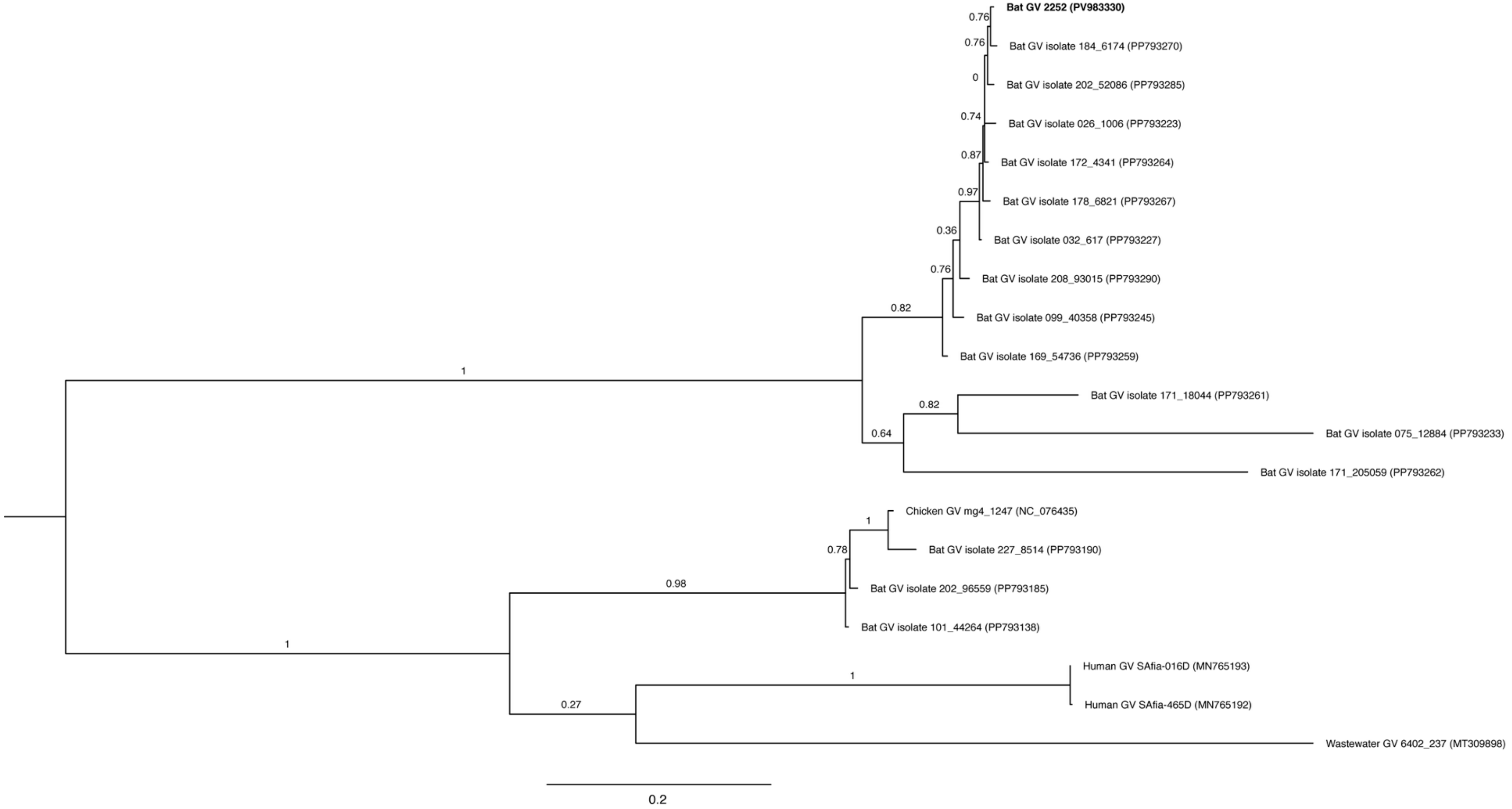

Supplement: Supplementary file 1 [file viruses-17-01349-s001.zip › viruses-3883665-supplementary.pdf]
